# Comparison of clinical features on admission between coronavirus disease 2019 and influenza a among children: a retrospective study in China

**DOI:** 10.1186/s12879-021-06037-3

**Published:** 2021-04-17

**Authors:** Feng Liang, Xianfeng Wang, Jianbo Shao, Jun Chen, Lei Liu, Hui Li, Yi Xu, Liya He, Huiying Liang, Kuanrong Li, Sitang Gong, Huimin Xia

**Affiliations:** 1 Clinical Data Center, Guangzhou Women and Children’s Medical Center, Guangzhou Medical University, Guangzhou, 510623 Guangdong China; 2 Department of Pediatric, The Third People’s Hospital of Shenzhen, Second Affiliated Hospital of Southern University of Science and Technology, Shenzhen, Guangdong China; 3 Department of Pediatric, Wuhan Children’s Hospital, Wuhan, 430000 Hubei China; 4 Department of Infectious Disease, The Third People’s Hospital of Shenzhen, Shenzhen, 518112 Guangdong China; 5 Department of Pediatric, Guangzhou Women and Children’s Medical Center, Guangzhou Medical University, Guangzhou, Guangdong China; 6 Guangdong Provincial Children’s Medical Research Center, Guangzhou Women and Children’s Medical Center, Guangzhou Medical University, Guangzhou, Guangdong China

**Keywords:** Coronavirus disease 2019, Influenza a, Pediatrics, Retrospective study

## Abstract

**Background:**

Coronavirus disease 2019 (COVID-19) share similar symptoms with influenza A (IA), but it is more worthwhile to understand the disparities of the two infections regarding their clinical characteristics on admission.

**Methods:**

A total of 71 age-matched pediatric IA and COVID-19 patient pairs were formed and their clinical data on admission were compared.

**Results:**

Fever, cough, nasal congestion and nausea/vomiting were the most common symptoms on admission for both infections but occurred less often in COVID-19. The IA patients were more likely to have lower-than-normal levels of lymphocyte count and percentage and to have higher-than-normal levels of activated partial thromboplastin time, prothrombin time, serum C-reactive protein, and serum procalcitonin, while the COVID-19 patients had higher odds of having lower-than-normal levels of neutrophil count and percentage.

**Conclusions:**

This study suggests that influenza A is more symptomatic than COVID-19 for children and might be an overall more severe infection at the time of admission.

**Supplementary Information:**

The online version contains supplementary material available at 10.1186/s12879-021-06037-3.

## Introduction

Severe acute respiratory syndrome coronavirus 2 (SARS-CoV-2) is a newly emerging member of the coronaviridae family causing the currently ongoing global pandemic of the coronavirus disease 2019 (COVID-19), which started in December 2019 in Wuhan, China and spread rapidly to many other parts of the world. At the time of writing, the total number of confirmed cases has climbed beyond the 57.2 million mark, with a death toll of more than 1368,000 people [1].

According to the data published at the beginning of this pandemic, SARS-CoV-2 causes disproportionally fewer COVID-19 s among young children than among adults, with no more than 1.0% of the confirmed cases falling into the age group of 0–9 years [2, 3]. Symptoms of COVID-19 in children are similar to those in adults but are in general milder and occur less often [3, 4].

Another virus that has potential to cause a similar global pandemic is influenza A (IA) virus. IA virus and SARS-CoV-2 cause similar symptoms such as fever and cough, especially at the early stages of the disease. However, contrary to COVID-19, studies have shown that IA manifests more symptoms in children than in adult [5]. Therefore, it is necessary to study the symptomological disparities between the two diseases in pediatric patients. Furthermore, the question whether the two infections are also different in their hematological and biochemical measurements needs to be answered.

In this report, we looked to examine whether COVID-19 and IA differ in their clinical characteristics on admission among 71 age-matched pediatric patient pairs that were diagnosed between September 2019 and February 2020.

## Material and methods

### Data sources

We initially included 106 pediatric COVID-19 cases hospitalized in Guangzhou Women and Children’s Medical Center or in The Third People’s Hospital of Shenzhen between January 23 to February 15, 2020, or in Wuhan Children’s Hospital between February 5 and 23, 2020. All the diagnoses were made following the then latest diagnosis and treatment protocol developed by the National Health Commission [6]. All the COVID-19 cases were laboratory-confirmed by real-time reverse transcriptase polymerase chain reaction (RT-PCR) and none of them had laboratory-confirmed co-infections of other respiratory viruses. Of our study population, the COVID-19 patients were also included in the analyses of other publications [7–9].

Following a case-control-like design, we searched the inpatient database of Guangzhou Women and Children’s Medical Center for pediatric IA patients who were admitted to the hospital between September 1, 2019 and February 23, 2020 and who were also RT-PCR confirmed. After excluding those with laboratory confirmed co-infections of other respiratory viruses, a selection pool of 296 patients was formed. From this pool, we selected an age-matched IA patient for each of the 106 COVID-19 patients, where an eligible match referred to an absolute difference in age at admission for a patient pair no greater than 30 days. To do so, we produced an exhaustive paring of the 106 COVID-19 patients and the 296 IA patients and started with a randomly selected COVID-19 patient, for whom the first pair with the minimum age difference was selected. If that COVID-19 patient happened to have multiple eligible age-matched IA patients, priority would be given to the one of the same sex. The IA patient included in the selected pair was then removed from the pool without replacement. This process was repeated for each of the remaining COVID-19 patients following a random sequence, eventually resulting in 71 COVID-19 and IA patient pairs that met the matching criterion.

Medical records of the 106 COVID-19 patients were retrieved from the three participating hospitals and were then centralized at Guangzhou Women and Children’s Medical Center, where eight data specialists extracted the clinical data using a standardized case report form under the supervision of two COVID-19 experts. Each of the medical records was reviewed by two data specialists, and inconsistencies and uncertainties raised during data extraction were solved by consulting with the COVID-19 experts or contacting the treating physicians for clarification.

For the age-matched IA patients, their clinical data were derived from electronic medical records by the same researchers and were handled the same way when further clarifications were required. The symptomological, hematological, and blood biochemical features at the time of hospital admission between the COVID-19 and the IA patients were compared.

This study was approved by the ethics committees of Guangzhou Women and Children’s Medical Center, The Third People’s Hospital of Shenzhen and Wuhan Children’s Hospital. Written informed consent was obtained from the parents or guardians of the COVID-19 patients. As IA patients were not recruited but retrieved from the inpatient care database, written informed consent was not required from them.

### Statistical analyses

For descriptive analysis, median and interquartile range were calculated for continuous data and percentages were calculated for categorical data. Abnormally high or low laboratory measurements were determined using the established reference ranges, which might be age-specific or universal for all age groups (Table S1). For inferential analysis, continuous data were compared with Wilcoxon signed-rank test. McNemar’s test was used to determine whether the percentages of the abnormally high or low measurement for a particular biomarker were statistically significantly different between the IA and the COVID-19 patients, which was denoted with the *P* value and the odds ratio as well as its 95% confidence interval. The statistical analyses were performed using the R software (Version 3.5.2, R Foundation, Vienna, Austria).

## Results

In the 71 age-matched patient pairs, boys accounted for 57.7% (41/71) for COVID-19 and 54.9% (39/71) for IA, and 83.1% (59/71) of the pairs were also matched on sex. For both the COVID-19 and the IA patients, the median age at admission was 2.6 years (rang: 0–13.8). The COVID-19 cases were diagnosed between January 23, 2020 and February 25, 2020, while the IA cases were diagnosed between September 1, 2019 and January 31, 2020.

**Fig. 1:**
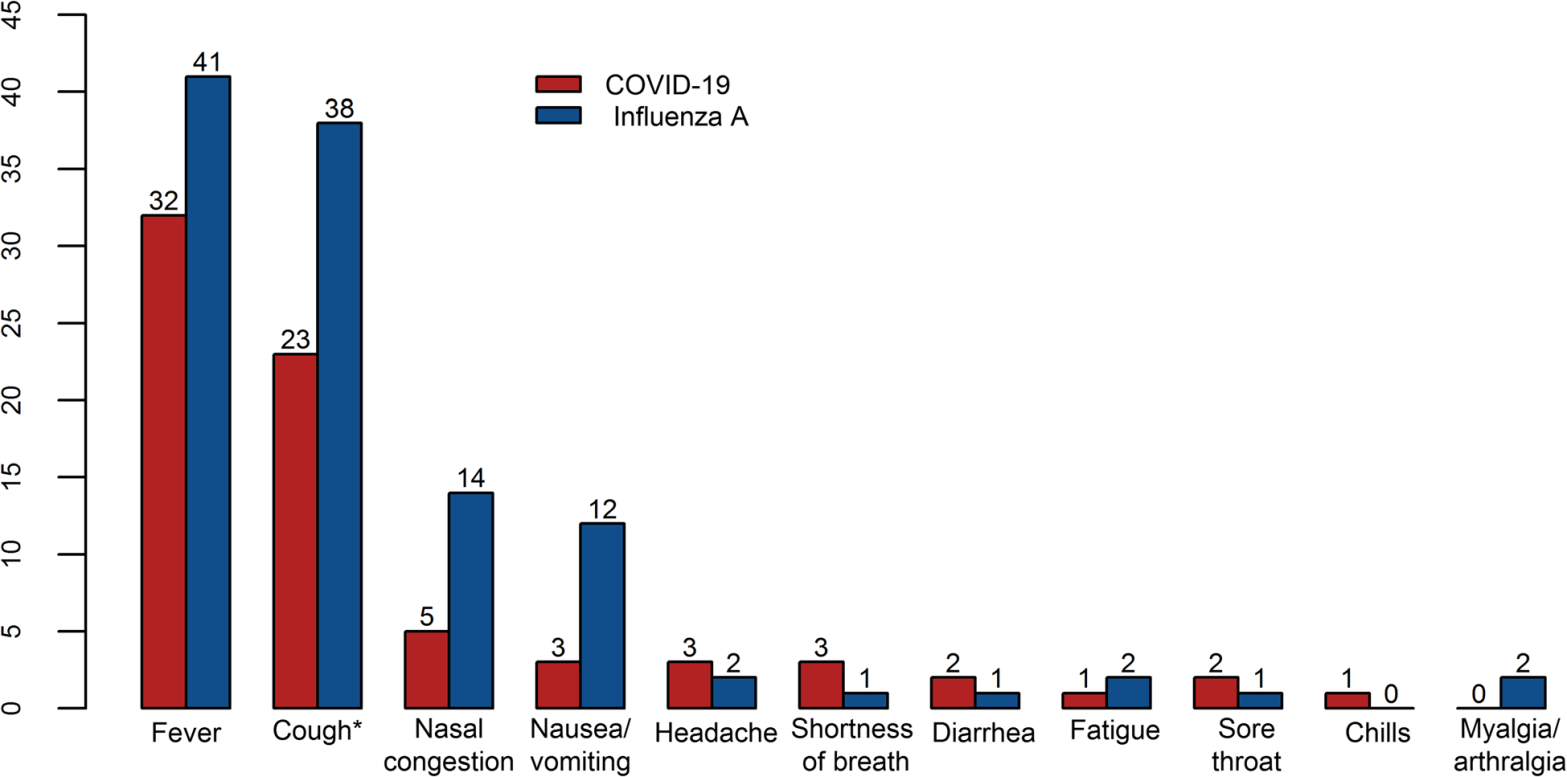
Distribution of symptoms on admission in 45 COVID-19 and influenza A patient pairs *Dry cough vs. wet cough: 14 vs. 9 for COVID-19 and 16 vs. 22 for influenza A

Among the 71 COVID-19 patients, 26 were admitted only because of a positive RT-PCR result after close exposure to infected individuals, most of whom were their parents or principle caregivers. When we compared the distributions of the symptoms on admission, this subgroup of patients and their IA pairs were excluded. Among the remaining, symptomatic patients (*n* = 45), fever (71.1% for COVID-19 and 91.1% for IA) and cough (51.1% for COVID-19 and 84.4% for IA) occurred much more often than other symptoms (Fig. 1). Fever, cough, nasal congestion, and nausea/vomiting were the top four most common symptoms for both diseases but were seen more frequently in the IA patients. The differences in the frequencies of these symptoms were all statistically significant, and the odds ratios of manifesting them for IA vs. COVID-19 varied from 4 to 10 (Table 1).

**Table 1:** Common symptoms on admission of the 45 age-matched pediatric IA and COVID-19 patient pairs who were symptomatic at admission^a^

	I_yes_C_yes_	I_yes_C_no_	I_no_C_yes_	I_no_C_no_	*P*	OR (95% CI)
Fever	31 (68.9)	10 (22.2)	1 (2.2)	3 (6.7)	0.02	10.00 (1.28, 78.12)
Cough	18 (40)	20 (44.4)	5 (11.1)	2 (4.4)	< 0.01	4.00 (1.50, 10.65)
Dry cough	6 (13.3)	10 (22.2)	8 (17.8)	21 (46.7)	0.81	1.25 (0.49, 3.17)
Wet cough	5 (11.1)	17 (37.8)	4 (8.9)	19 (42.2)	< 0.01	4.25 (1.43,12.63)
Nasal congestion	2 (4.4)	12 (26.7)	3 (6.7)	28 (62.2)	0.04	4.00 (1.13, 14.17)
Nausea or vomiting	1 (2.2)	11 (24.4)	2 (4.4)	31 (68.9)	0.03	5.50 (1.22, 24.81)

^a^Data are presented in count and percentage (in parentheses). The *P* values were calculated using McNemar’s χ^2^ test. The I and C combinations denote whether a particular symptom was present (subscript yes) or not (subscript no) for an IA (I) and COVID-19 (C) patient pair

*Abbreviations*: *COVID-19* coronavirus disease 19, *CI* confidence interval, *IA* influenza A, *OR* odds ratio

Laboratory tests were carried out for 99% of the IA patients and 97% of the COVID-19 patients within the first 48 h after admission. In comparison to the COVID-19 patients, the IA patients had lower levels of lymphocyte count, lymphocyte percentage, neutrophil-to-lymphocyte ratio (NLR), haemoglobin (*n* = 59), and albumin (*n* = 61) but higher levels of neutrophil count, neutrophil percentage (n = 59), activated partial thromboplastin time (APTT), prothrombin time (PT) (*n* = 51), C-reactive protein(n = 61), and procalcitonin (*n* = 44). All these differences were statistically significant (Table 2) and remained statistically significant after limiting the analysis to symptomatic patients (Table S2). Data of other hematological and biochemical measurements showing no statistically significant difference are reported in Table S3.

**Table 2:** Hematological and blood biochemical measurements of the 71 age-matched pediatric IA and COVID-19 patient pairs^a^

	IA	COVID-19	*P*
**Blood routine(*n*** **= 59)**			
Lymphocytes			
Count(×10^9^/L)	1.8 (1.0–3.4)	3.2 (1.8–5.0)	< 0.01
Percentage (%)	26.0 (12.0–44.0)	50.4 (36.6–68.6)	< 0.01
Neutrophils			
Count (×10^9^/L)	4.2 (2.8–7.7)	2.2 (1.4–4.1)	< 0.01
Percentage (%)	62.0 (43.0–78.0)	37.7 (21.5–49.1)	< 0.01
Hemoglobin (g/L)	112.0 (101.0–123.0)	123.0 (111.0–128.0)	< 0.01
Neutrophil-to-lymphocyte ratio	2.6 (0.9–7.1)	0.7 (0.3–1.4)	< 0.01
**Coagulation function(n = 51)**			
Activated partial thromboplastin time (s)	42.4 (38.7–46.3)	35.2 (30.9–40.5)	< 0.01
Prothrombin time (s)	14.0 (12.7–15.1)	11.6 (10.7–12.8)	< 0.01
**Blood biochemistry**			
Albumin (g/L) (n = 61)	42.0 (39.9–43.6)	44.2 (42.1–46.7)	< 0.01
C-reactive protein (mg/L) (n = 61)	5.8 (1.2–20.6)	0.9 (0.8–5.0)	< 0.01
Procalcitonin (ng/mL) (n = 44)	0.4 (0.2–1.0)	0.1 (0.1–0.1)	< 0.01

^a^For each measurement, the exact number of patient pairs included in the analysis varied due to missing values. Distribution of the measurements is denoted by median and interquartile range (in parentheses). The *P* values were calculated using Wilcoxon signed-rank test

*Abbreviations*: *COVID-19* coronavirus disease 19, *IA* influenza A

As shown in Table 3, the IA patients had higher odds of having lower-than-normal levels of lymphocyte count, lymphocyte percentage, and haemoglobin, with odds ratios being 5.50, 9.50, and 6.50, respectively, while the COVID-19 patients had higher odds of having abnormally low levels of neutrophil count and percentage. The IA patients also had higher odds of having abnormally high APTT and PT. With respect to blood biochemical markers, abnormally high serum C-reactive protein and procalcitonin levels occurred with higher odds in the IA patients. Data for other markers showing statistically non-significant results are given in Table S4.

**Table 3:** Hematological and blood biochemical measurements of the 71 age-matched pediatric IA and COVID-19 patient pairs: abnormally high or low^a^

	I_yes_C_yes_	I_yes_C_no_	I_no_C_yes_	I_no_C_no_	*P*	OR (95% CI)
**Blood routine (n = 59)**						
Lymphocytes						
Count abnormally low	5 (8.5)	22 (37.3)	4 (6.8)	28 (47.5)	< 0.01	5.50 (1.90–16.0)
Percentage abnormally low	3 (5.1)	19 (32.2)	2 (3.4)	35 (59.3)	< 0.01	9.50 (2.21–40.8)
Neutrophils						
Count abnormally low	4 (6.8)	2 (3.4)	19 (32.2)	34 (57.6)	< 0.01	0.11 (0.02–0.45)
Percentage abnormally low	0 (0)	0 (0)	6 (10.2)	53 (89.8)	0.03	0.00 (0.00–0.85)
Hemoglobin						
Abnormally low	4 (6.8)	13 (22.0)	2 (3.4)	40 (67.8)	< 0.01	6.50 (1.47–28.8)
**Coagulation function (n = 51)**						
APPT (s) †						
Abnormally high	2 (3.9)	18 (35.3)	0 (0)	31 (60.8)	< 0.01	–
PT (s) †						
Abnormally high	0 (0)	13 (25.5)	0 (0)	38 (74.5)	< 0.01	–
**Blood biochemistry**						
C-reactive protein (n = 61)						
Abnormally high	5 (8.2)	24 (39.3)	7 (11.5)	25 (41.0)	< 0.01	3.43 (1.48–7.96)
Procalcitonin (n = 44)						
Abnormally high	10 (22.7)	28 (63.6)	3 (6.8)	3 (6.8)	< 0.01	9.33 (2.84–30.7)

^a^For each measurement, the exact number of patient pairs included into the analysis varied due to missing values. Data are presented in count and percentage (in parentheses). The *P* values were calculated using McNemar’s χ^2^ test. The I and C combinations denote whether a particular measurement was abnormally high/low (subscript yes) or not (subscript no) for an IA (I) and COVID-19 (C) patient pair. †OR was not calculable due to the zero value for the I_no_C_yes_ combination

*Abbreviations*: *APTT* activated partial thromboplastin time, *COVID-19* coronavirus disease 19, *CI* confidence interval, *IA* influenza A, *OR* odds ratio, *PT* prothrombin time

Among the 71 COVID-19 patients, 69 received thoracic computerized tomography (TCT) scan on admission, revealing ground-glass opacity in 14 cases, bilateral patchy shadowing in 13 cases, and unilateral patchy shadowing in 19 cases. A total of 12 patients out of the 14 cases with ground-class opacity also manifested bi/unilateral patchy shadowing. In contrast, TCT scan was administered to only nine of the 71 IA patients, with one showing ground-glass opacity, three showing unilateral patchy shadowing, and three showing bilateral patchy shadowing). Forty-nine of the IA patients nevertheless underwent Chest X-ray examination: none of them manifested ground-class opacity and 15 manifested either unilateral or bilateral patchy shadowing.

At the time of data extraction, thirty COVID-19 patients remained hospitalized, the others had been discharged alive, and only one patient had been admitted to ICU and put on ventilator due to acute respiratory distress syndrome. Among the 71 IA patients, four were admitted to ICU for mechanical ventilation and no death occurred.

## Discussion

By comparing the clinical characteristics on admission of 71 pairs of age-matched pediatric IA patients and COVID-19 patients, this study showed that typical IA symptoms were also the most common symptoms for the COVID-19 patients regardless of their relatively rarer occurrence. The levels of some hematological and blood biochemical measurements differed between the IA and COVID-19 patients: abnormally low levels of lymphocyte count and percentage, and abnormally high levels of APTT, PT, C-reactive protein, and procalcitonin were seen more often in the IA patients, while abnormally low levels of neutrophil count and percentage occurred more often in the COVID-19 patients.

The present study confirms the previous reports that fever and cough were the most common symptoms for pediatric COVID-19 patients [3, 7, 10, 11]. In the present study, fever and cough occurred in 71 and 51% of the COVID-19 patients, respectively. In a larger study of 291 U.S. pediatric COVID-19 patients, the corresponding figures were 56 and 54%, respectively [3]. It should be noted that the present study excluded asymptomatic patients in order to make a fair comparison between them and the IA patients who presented to hospital with definite symptoms. Relatively lower occurrences of clinical symptoms have been reported by previous studies, where an increasing proportion (varying from 4.4 to 27.8%) of asymptomatic but laboratory confirmed patients were included as a result of the continuously expanding coverage of the RT-PCR screening among asymptomatic individuals [12–14].

IA and COVID-19 are two infections sharing similar spectrums of symptoms. In addition, studies have shown that these symptoms occur in similar frequencies in adult IA and COVID-19 patients [3, 5, 12, 15, 16]. In this study of pediatric patients, however, COVID-19 caused fewer symptoms in spite of the exclusion of the asymptomatic cases. This finding, together with the data from other studies [3, 4], suggests that adult COVID-19 patients might be more symptomatic than their pediatric counterparts.

Lymphopenia has been found to be a common feature of IA infection for both children and adults and to occur in more than 90% of the pediatric patients [17–19]. However, it seemed not the case for children with COVID-19, given its low incidence (9/59) seen in this study. In another study, lymphopenia occurred only in 3.5% of the pediatric patients of COVID-19, partly due to its stricter definition of lymphopenia (< 1.2 × 10^9^/L) [4]. A recent pooled analysis of more than 1000 adult COVID-19 patients suggests an association between decreased lymphocyte count and intensive care unit (ICU) stay [20]. Data from the present study supports this association in a way that only nine of the 71 COVID-19 patients had abnormally low lymphocyte count and only one patient was eventually admitted to ICU. Previous studies have suggested that excessive neutrophil activation correlates with worsening oxygenation impairment and predicted fatal outcome in both COVID-19 patients and IA patients [21–23]. The higher levels of neutrophils count and neutrophils percentage among the IA patients suggest that IA might be more severe and prone to poorer diagnosis. Neutropenia has been reported to have an incidence of 10% in adult IA patients [19], but it is unclear how often it occurs in pediatric patients and whether it has an association with the disease prognosis. For adult COVID-19 patients, a small study showed a higher level of neutrophil count in patients admitted to ICU than in those not [16]. NLR is useful in diagnosing influenza virus infections and in discriminating other respiratory infections [24, 25]. In COVID-19 patients, elevated NLR is an independent factor for poor clinical outcome [26, 27]. In this article, IA patients showed a higher level of NLR compared with COVID-19 patients, suggesting a potential difference in severity between the two infections.

By activating the coagulation system via complex mechanisms including the mediation of cytokines, viral infections can lead to aberrant hemostasis [28]. As a result, prolonged APTT and PT have been observed in both severe IA and COVID-19 [29, 30]. Thus, the averagely higher levels of PT and APTT and the higher proportions of higher-than-normal APTT an PT cases among the IA patients suggest that IA might be overall more severe and prone to poorer diagnosis.

Among the IA patients, we also observed higher proportions of cases with abnormally high levels of C-reactive protein and procalcitonin, which are both key inflammatory markers and are associated with poor prognosis such as pneumonia and even death [31–35]. To date, data from small studies also suggest a positive association between the level of C-reactive protein and the severity of COVID-19 [36, 37]. Elevated procalcitonin level has been observed for 30–80% of pediatric patients in separate studies, based on different thresholds [4, 10, 11]. In one of these studies, the level of procalcitonin was higher in patients complicated with pneumonia than in patients with upper respiratory tract infection and patients who were asymptomatic [4]. Once again, our results regarding these two markers support that IA is likely to be a more severe infection for children than COVID-19.

In this study, the levels of other hematological and biochemical measurements than the ones we mentioned above showed no statistically significant difference between the two infections. Among them, we noticed that abnormally low platelet count, abnormally high alanine aminotransferase, and abnormally high level of aspartate aminotransferase occurred more often in the IA patients, which might show statistically significance if the sample size increased. Elevation of these measurements are commonly seen in IA patients [15], but whether the different levels of these measurements indicate different disease severity warrants further investigations.

It is known that COVID-19 shares symptomological similarities with IA, but it might be more worthwhile to detail the disparities between the two infections, as doing so provides health professionals with important information regarding how to handle this new disease via sensible utilization of their existing knowledge and experience in managing IA patients. This study, to the best of our knowledge, has been the first study that serves this purpose. This study benefited from its age-matching design and the use of age-specific clinical thresholds for hematological and biochemical measurements. However, given its small sample size, the results of this study need to be confirmed by larger studies. Also, COVID-19 and IA might display distinct imaging manifestations among children, but only nine out of the 71 IA patients received thoracic TCT scan, making a meaningful comparison between the two diseases impossible. Lastly, because of lack of reliable definitions of disease severity and the incompleteness of prognosis data for the COVID-19 patients, we could not further examine whether the differed clinical characteristics on admission of the two infections eventually lead to different prognosis.

## Conclusion

In conclusion, based on the disparities in the clinical manifestations on admission, this study suggests influenza A is a more symptomatic infection than COVID-19 for children and might also be a more severe infection at the time of admission.

## Supplementary Information

**Additional file 1.**

## Data Availability

The datasets used and/or analysed during the current study are available from the corresponding author on reasonable request.

## Abbreviations

COVID-19: Coronavirus disease 2019
IA: Influenza A
CI: Confidence interval
APTT: Activated partial thromboplastin time
OR: Odds ratio
PT: Prothrombin time
RT-PCR: Real-time reverse transcriptase polymerase chain reaction
SARS-CoV-2: Severe acute respiratory syndrome coronavirus 2
TCT: Thoracic computerized tomography
