# Supplementary material for: Comparison of clinical features on admission between coronavirus disease 2019 and influenza a among children: a retrospective study in China
Source: BMC Infect Dis. 2021 Apr 17;21:365. doi: 10.1186/s12879-021-06037-3 (PMC8052833; doi:10.1186/s12879-021-06037-3)
Supplement: Supplementary file 1 — Additional file 1. [file 12879_2021_6037_MOESM1_ESM.docx]

**Table S1. Reference ranges for hematological and blood biochemical measurements used in this study. ^a^**

|  | 0 -14 d | 15-30 d | 31d<0.5 y | 0.5-5.9 y | 6-17.9 y |
| --- | --- | --- | --- | --- | --- |
| Leucocyte count (×10^9^/L) | 4.94-27.48 | 7.80-15.91 | 6.00-14.99 | 4.86-13.51 | 3.84-11.4 |
| Platelet count (×10^9^/L) | 144-450 | 248-586 | 229-597 | 189-459 | 175-369 |
| Lymphocyte percentage (%) | 24.9-68.5 | 31.9-82.7 | 30.4-86.7 | 18.1-79.9 | 15.5-57.8 |
| Neutrophil percentage (%) | 15.2-66.1 | 10.6-57.3 | 8.9-76.0 | 16.9-74.0 | 28.6-74.7 |
| Lymphocyte count (×10^9^/L) | 1.55-4.80 | | | | |
| Neutrophil count (×10^9^/L) | 2.0-7.2 | | | | |
| Hemoglobin (g/L) | 105-145 | | | | |
| Activated partial thromboplastin time (s) | 28-45 | | | | |
| Prothrombin time (s) | 11-15 | | | | |
| Albumin (g/L) | 40–55 | | | | |
| Alanine aminotransferase (U/L) | 9–50 | | | | |
| Aspartate aminotransferase (U/L) | 5-60 | | | | |
| Total bilirubin (μmol/L) | 2–17 | | | | |
| Blood urea nitrogen (mmol/L) | 2.1-7.1 | | | | |
| Serum creatinine (μmol/L) | 18-62 | | | | |
| Creatine kinase (U/L) | 45-390 | | | | |
| Lactate dehydrogenase (U/L) | 159-322 | | | | |
| C-reactive protein (mg/L) | 0-6 | | | | |
| Procalcitonin (ng/mL) | 0-0.1 | | | | |

**Notes:** ^a^The reference ranges for leucocyte count, platelet count, lymphocyte percentage, and neutrophil percentage are age-specific and those for the others are universal for all age groups.

**Table S2. Hematological and blood biochemical measurements of the 45 age-matched pediatric IA and COVID-19 patient pairs. ^a^**

|  | IA | COVID-19 | *P* |
| --- | --- | --- | --- |
| **Blood routine (n=37)** |  |  |  |
| Lymphocytes |  |  |  |
| Count (×10^9^/L) | 2.1 (0.9-4.1) | 3.6 (1.9-5.0) | <0.01 |
| Percentage (%) | 26.0 (12.5-46.0) | 50.4 (40.9-68.0) | <0.01 |
| Neutrophils |  |  |  |
| Count (×10^9^/L) | 4.5 (2.7-7.4) | 2.2 (1.5-4.4) | <0.01 |
| Percentage (%) | 63.0 (40.5-77.0) | 35.8 (21.3-48.1) | <0.01 |
| Hemoglobin (g/L) | 112.0 (100.5-121.5) | 116.0 (110.5-128.0) | 0.01 |
| Neutrophil-to-lymphocyte ratio | 2.6 (0.9-7.2) | 0.7 (0.3-1.3) | <0.01 |
| **Coagulation function(n=33)** |  |  |  |
| Activated partial thromboplastin time (s) | 44.6 (38.5-47.7) | 34.5 (30.1-40.3) | <0.01 |
| Prothrombin time (s) | 13.8 (12.7-15.0) | 11.4 (10.7-12.5) | <0.01 |
| **Blood biochemistry** |  |  |  |
| Albumin (g/L) (n=41) | 42.0 (40.1-43.9) | 44.1 (41.1-46.2) | 0.01 |
| C-reactive protein (mg/L) (n=37) | 5.9 (1.7-30.0) | 1.0 (0.8-6.4) | 0.05 |
| Procalcitonin (ng/mL) (n=29) | 0.31 (0.16,0.83) | 0.09 (0.06,0.19) | <0.01 |

**Notes:** ^a^For each measurement, the exact number of patient pairs included in the analysis varied due to missing values. Distribution of the measurements is denoted by median and interquartile range (in parentheses). The *P* values were calculated using Wilcoxon signed-rank test.

**Abbreviations:** COVID-19, coronavirus disease 19; IA, influenza A.

**Table S3. Other hematological and blood biochemical measurements of the 71 age-matched pediatric IA and COVID-19 patient pairs. ^a^**

|  | IA | COVID-19 | *P* |
| --- | --- | --- | --- |
| **Blood routine(n=59)** |  |  |  |
| Leucocyte count (×10^9^/L) | 8.6(5.4-10.9) | 7.2(5.5-9.9) | 0.11 |
| Platelet count (×10^9^/L) | 261.0(207.0-328.0) | 286.0(232.0-376.0) | 0.26 |
| **Blood biochemistry** |  |  |  |
| Alanine aminotransferase (U/L) (n=64) | 18.0(13.0-28.0) | 19.9(12.0-30.5) | 0.43 |
| Aspartate aminotransferase (U/L) (n=64) | 39.5(32.3-45.8) | 40.5(31.5-51.0) | 0.81 |
| Total bilirubin (μmol/L) (n=61) | 4.3(2.9-7.3) | 6.3(4.1-9.5) | 0.05 |
| Lactate dehydrogenase (U/L) (n=62) | 286.5(241.0-342.3) | 281.5(230.8-359.5) | 0.16 |
| Blood urea nitrogen (mmol/L) (n=40) | 3.5(2.8-4.3) | 3.7(2.4-4.9) | 0.60 |
| Serum creatinine (μmol/L) (n=63) | 26.0(22.0-32.0) | 26.8(23.0-35.0) | 0.56 |
| Creatine kinase (U/L) (n=59) | 122.0(79.0-187.0) | 99.0(80.0-152.0) | 0.26 |
|  | | | |

**Notes:** ^a^For each measurement, the exact number of patient pairs included in the analysis varied due to missing values. Distribution of the measurements is denoted by median and interquartile range (in parentheses). The *P* values were calculated using Wilcoxon signed-rank test.

**Abbreviations:** COVID-19, coronavirus disease 19; IA, influenza A.

**Table S4. Other hematological and blood biochemical measurements of the 71 age-matched pediatric IA and COVID-19 patient pairs: abnormally high or low^a^**

|  | I_yes_ C_yes_ | I_yes_ C_no_ | I_no_C_yes_ | I_no_C_no_ | *P* | OR (95% CI) |
| --- | --- | --- | --- | --- | --- | --- |
| **Blood routine(n=59)** |  |  |  |  |  |  |
| Leucocyte count |  |  |  |  |  |  |
| Abnormally low | 1 (1.7) | 4 (6.8) | 4 (6.8) | 50 (84.7) | 1.00 | 1.00 (0.19, 5.37) |
| Platelet count |  |  |  |  |  |  |
| Abnormally low | 5 (8.5) | 8 (13.6) | 4 (6.8) | 42 (71.2) | 0.39 | 2.00 (0.60, 6.64) |
| **Blood biochemistry** |  |  |  |  |  |  |
| Albumin (n=61) |  |  |  |  |  |  |
| Abnormally low | 5 (8.2) | 10 (16.4) | 6 (9.8) | 40 (65.6) | 0.45 | 1.67 (0.61, 4.59) |
| Alanine aminotransferase (n=64) |  |  |  |  |  |  |
| Abnormally high | 0 (0) | 5 (7.8) | 3 (4.7) | 56 (87.5) | 0.72 | 1.67 (0.32, 10.7) |
| Aspartate aminotransferase (n=64) |  |  |  |  |  |  |
| Abnormally high | 0 (0) | 10 (15.6) | 7 (10.9) | 47 (73.4) | 0.63 | 1.43 (0.54, 3.75) |
| Total bilirubin (n=61) |  |  |  |  |  |  |
| Abnormally high | 2 (3.3) | 3 (4.9) | 0 (0) | 56 (91.8) | 0.25 | - |
| Lactate dehydrogenase (n=62) |  |  |  |  |  |  |
| Abnormally high | 10 (16.1) | 9 (14.5) | 13 (21.0) | 30 (48.4) | 0.52 | 0.69 (0.30, 1.62) |
| Blood urea nitrogen (n=40) |  |  |  |  |  |  |
| Abnormally high | 0 (0) | 1 (2.5) | 0 (0) | 39 (97.5) | 1.00 | - |
| Serum creatinine (n=63) |  |  |  |  |  |  |
| Abnormally high | 0 (0) | 0 (0) | 2 (3.2) | 61 (96.8) | 0.50 | 0.00 (0.00, 5.32) |
| Creatine kinase (n=59) |  |  |  |  |  |  |
| Abnormally high | 1 (1.7) | 6 (10.2) | 0 (0) | 52 (88.1) | 0.04 | - |

**Notes:** ^a^For each measurement, the exact number of patient pairs included into the analysis varied due to missing values. Data are presented in count and percentage (in parentheses). The *P* values were calculated using McNemar's χ² test. The I and C combinations denote whether a particular measurement was abnormally high/low (subscript yes) or not (subscript no) for an IA (I) and COVID-19 (C) patient pair. †OR was not calculable due to the zero value for the I_no_C_yes ­_combination.

**Abbreviations:** COVID-19, coronavirus disease 19; CI, confidence interval; IA, influenza A; OR, odds ratio.
